# Kinetochore-microtube attachments in cancer therapy

**DOI:** 10.18632/oncoscience.265

**Published:** 2015-11-16

**Authors:** Donatella Del Bufalo, Francesca Degrassi

**Affiliations:** Institute of Molecular Biology and Pathology, National Research Council, Rome, Italy

**Keywords:** chromosome instability, kinetochore-microtubule attachment

**Figure 1:**
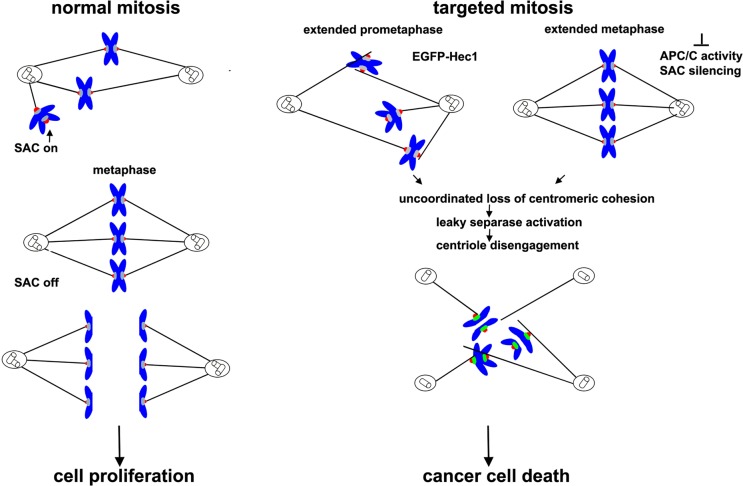
Induction of multipolarity by interfering with kinetochore-microtubule attachment dynamics as an anticancer strategy Left panel. During the initial stages of a normal mitosis, unattached kinetochores activate the spindle assembly checkpoint, halting anaphase onset. At metaphase, each sister kinetochore interacts with microtubules coming from a different pole so that the spindle assembly checkpoint is silenced and an accurate chromosome segregation occurs. Right panel. When kinetochore-microtubule attachment dynamics is perturbed (EGFP-Hec1 extended prometaphase or extended metaphase), mitotic cells remain for longer time period in prometaphase or metaphase. This promotes an uncoordinated loss of centromeric cohesion and a partial separase activation that leads to the splitting of the centrioles and to the formation of multipolar spindles. Cells then die in mitosis or in the following interphase as a result of a massive chromosome missegregation.

The process of cell division represents an extraordinary target to develop antitumor therapies. Indeed, a large number of clinically relevant anti-cancer drugs, such as taxanes and vinca alkaloids, target mitosis by stimulating or inhibiting microtubule (MT) polymerization. During the past decades anti-tubulin drugs have proven very effective against a wide range of tumors. However, collateral effects, such as myelosuppression and MT disruption in non-dividing tissues, including brain, are common. Recently, the increased understanding of the cell division process and the identification of several signaling pathways controlling mitosis have provided novel opportunities for cancer drug discovery. Consequently, mitotic proteins have become attractive targets to develop molecular cancer therapeutics. In this scenario, kinetochores (KTs) represent an attractive therapeutic target in light of their fundamental role in driving chromosome segregation and controlling chromosome segregation errors. Indeed, cells require a fine regulation of the kinetochore-microtubule (KT-MT) attachment stability to prevent chromosome instability, and KT-MT attachment dynamics is often deregulated in tumour cells [2]. Chromosome instability is commonly accepted as a driving force in the development of cancer, but more recent work has demonstrated that extensive chromosome missegregation may be detrimental to cancer cells and act as a tumor suppression mechanism [3]. In light of this double role of chromosome instability in cancer, we have explored the hypothesis that interfering with KT-MT attachment dynamics could drive massive chromosome missegregation and kill tumor cells. Highly Expressed in Cancer protein 1 (Hec1) is a constituent of the evolutionary conserved Ndc80 complex, the molecular connector between KTs and MTs. Among the subunits of the Ndc80 complex, Hec1 directly interacts with MTs and regulates KT-MT dynamics and attachment stability [3]. Importantly, Hec1 is frequently overexpressed in cancer. We previously demonstrated that expression of Hec1 fused with the enhanced green fluorescent protein (EGFP) tag at its N-terminus (EGFP-Hec1), the protein domain that regulates MT attachment dynamics, led to a strong accumulation of this modified protein, which acted as a dominant negative mutant over the endogenous Hec1. Mitotic cells expressing a N-terminus tagged Hec1 accumulated lateral KT-MT attachments and underwent a spindle assembly checkpoint (SAC) dependent mitotic arrest associated with the formation of multipolar spindles [4]. We further showed that expression of an inducible N-terminus modified Hec1 completely abolished *in vitro* growth of EGFP-Hec1 expressing HeLa cells but had no effects on untransformed human fibroblasts or epithelial cells [5]. These *in vitro* cell-based data were validated *in vivo* by showing that inducible EGFP-Hec1 expression strongly inhibited tumor growth in a HeLa xenograft mouse model [5]. Strikingly, in both *in vitro* and *in vivo* models, EGFP-Hec1 expressing cells were permanently arrested in mitosis and produced multipolar spindles. Live imaging of EGFP-Hec1 expressing cells demonstrated that impaired chromosome segregation within multipolar spindles induced mitotic catastrophe, identified by the induction of apoptotic death from mitosis, or cytokinesis failure and multinucleation. Finally, measurements of MT flux rates and turnover at KT demonstrated that EGFP-Hec1 increased KT-MT attachment stability, suggesting that stabilizing KT-MT attachment dynamics represents a promising therapeutic approach [5]. Consistent with KT-MT attachment dynamics being the molecular target of the anticancer effect, expression of Hec1 fused with EGFP at its C-terminus, which does not affect KT-MT attachment dynamics, did not significantly affect cancer cell proliferation [5]. Collectively, our results demonstrate that massive chromosome missegregation within multipolar spindles can be used to kill tumor cells by activating a mitotic catastrophe process. In our experimental model, induction of multipolarity is caused by the extended time cells spend in prometaphase, which promotes cohesion fatigue (uncoordinated centromeric cohesion release) and concomitant centriole disengagement by leaky separase activation as depicted in Figure 1 [6,7]. Cancer cell death induced by cohesion fatigue-dependent multipolarity has been demonstrated following depletion of proteins controlling SAC silencing or after inhibition of the Anaphase Promoting Complex/cdc20 (APC/C) activity (Figure 1) and some of these treatments have been found more efficient than MT inhibitors in avoiding mitotic slippage and producing cancer cell death (7,8). These studies, together with our work, demonstrate that stimulation of spindle multipolarity can be used as an anti-cancer strategy through the activation of mitotic catastrophe after a multipolar mitosis. Moreover, they indicate that targeting the machineries involved in the regulation of KT-MT attachment dynamics, in the correction of KT-MT misattachments or in the silencing of the spindle assembly checkpoint may be a new frontier in the development of anticancer strategies.
