# Erratum: Kinetochore-microtube attachments in cancer therapy

**DOI:** 10.18632/oncoscience.291

**Published:** 2016-02-04

**Authors:** Donatella Del Bufalo, Francesca Degrassi

Oncoscience. 2015 Nov 16;2(11):902-3

***PMCID:*** *PMC4675780* ***PMID:*** *26697517*

***DOI:*** *10.18632/oncoscience.265*

In this Editorial, part of the title was incorrect.

The authors apologize for this oversight, it is corrected as follows.

Corrected title:

**Kinetochore-microtubule attachments in cancer therapy**

